# Strengthening systems for communicable disease surveillance: creating a laboratory network in Rwanda

**DOI:** 10.1186/1478-4505-9-27

**Published:** 2011-06-24

**Authors:** Senait Kebede, John B Gatabazi, Pierre Rugimbanya, Therese Mukankwiro, Helen N Perry, Wondimagegnehu Alemu, Jean B Ndihokubwayo, Michael H Kramer, Odette Mukabayire

**Affiliations:** 1 International Health Consultancy, Atlanta, USA; 2 Ministry of Health, National Reference Laboratory, Kigali, Rwanda; 3 Centers for Disease Control and Response, 1600 Clifton Rd., Atlanta, USA; 4 World Health Organization Regional Headquarters for Africa (AFRO), Brazzaville, Republic of Congo; 5 Ministry of Health, TRAC Plus, Kigali, Rwanda

## Abstract

**Background:**

The recent emergence of a novel strain of influenza virus with pandemic potential underscores the need for quality surveillance and laboratory services to contribute to the timely detection and confirmation of public health threats. To provide a framework for strengthening disease surveillance and response capacities in African countries, the World Health Organization Regional Headquarters for Africa (AFRO) developed Integrated Disease Surveillance and Response (IDSR) aimed at improving national surveillance and laboratory systems. IDSR emphasizes the linkage of information provided by public health laboratories to the selection of relevant, appropriate and effective public health responses to disease outbreaks.

**Methods:**

We reviewed the development of Rwanda's National Reference Laboratory (NRL) to understand essential structures involved in creating a national public health laboratory network. We reviewed documents describing the NRL's organization and record of test results, conducted site visits, and interviewed health staff in the Ministry of Health and in partner agencies. Findings were developed by organizing thematic categories and grouping examples within them. We purposefully sought to identify success factors as well as challenges inherent in developing a national public health laboratory system.

**Results:**

Among the identified success factors were: a structured governing framework for public health surveillance; political commitment to promote leadership for stronger laboratory capacities in Rwanda; defined roles and responsibilities for each level; coordinated approaches between technical and funding partners; collaboration with external laboratories; and use of performance results in advocacy with national stakeholders. Major challenges involved general infrastructure, human resources, and budgetary constraints.

**Conclusions:**

Rwanda's experience with collaborative partnerships contributed to creation of a functional public health laboratory network.

## Background

Communicable diseases remain the leading cause of illness, death and disability in African countries [1,2]. Even though well-known, efficacious responses are available for the control and prevention of these diseases, the capacity for timely detection, confirmation and response actions needs reliable public health systems. To address the demand from countries for improved surveillance systems that provide relevant and accurate epidemiologic and laboratory information, the Member States of the World Health Organization (WHO) Regional Headquarters for Africa (AFRO) adopted a strategy in 1998 called Integrated Disease Surveillance and Response (IDSR) [2]. A major goal of IDSR is to strengthen district-level surveillance capacities for detecting, confirming and responding to priority diseases that afflict African communities. In the IDSR implementation framework, epidemiologic surveillance is linked with laboratory support in order to produce relevant information for taking public health action [3].

The World Health Organization (WHO) emphasized the role of public health laboratories in national surveillance systems through a resolution in 2008 which called for the organization of national public health laboratory networks that would link national laboratories with sub-regional, regional and international laboratories [4,5]. Thus a public health laboratory network is a collection of laboratories that use standard operating procedures, carry out quality assessments, and report information for public health action in a systematic manner [4].

With the adoption of the revised International Health Regulations (2005) and the commitment of countries to achieve disease reduction targets set by the Millennium Development Goals (MDGs), countries are facing increased demands to rapidly improve their public health systems. The IHR (2005) are especially clear in calling for strengthening core capacities for detecting and confirming public health threats, especially those with potential to extend beyond national borders [6]. Thus progress in countries with limited resources depends on identifying opportunities for creating and streamlining resources to achieve functional national laboratory networks that meet national priorities and support global disease program objectives [7].

Disease-specific guidelines provide robust recommendations to create laboratory networks, but may not always include guidance on how to organize, mobilize and integrate the essential resources, procedures, and policies for creating and maintaining a laboratory network within a national public health surveillance system [8,9]. Thus we wanted to examine Rwanda's eight-year (from 2000 to 2007) experience with IDSR to see how the country developed a public health laboratory network through coordination of multiple resources and technical support in order to meet national priorities and partner interests.

In 2005, the Rwandan Ministry of Health commissioned an independent assessment of the national laboratory network to determine its status in relation to the national reference laboratory and for disease prevention programs [10]. Subsequently, in 2006, a team from WHO/AFRO, the United States Centers for Disease Control and Prevention (CDC) and the Rwanda National Reference Laboratory (NRL) in the Ministry of Health met to review Rwanda's public health laboratory network. The intention was to use the review findings to inform guidance for other countries that would make explicit practical factors to consider when implementing a public health laboratory network.

## Methods

A multi-agency team reviewed published and unpublished reports related to the implementation of Rwanda's laboratory activities, conducted site visits to observe how the laboratories functioned, and interviewed staff at the Ministry of Health and partner agencies. The National Reference Laboratory (NRL) coordinated the site visits to seven district hospitals and two health centers. These sites were selected as a convenience sample because each health center and district hospital in Rwanda offers the same standardized group of laboratory tests as specified by government policy. Interviews and follow-up discussions were conducted with laboratory personnel in charge of the district hospital and health center laboratories. Interviewees included district supervisors and representatives of disease-specific programs for tuberculosis (TB), malaria, and HIV/AIDS. We also met with personnel at the Ministry of Health's Epidemiology and Health Prevention unit, and with staff responsible for the Health Management Information System. Experiences with external support to laboratory services were gathered through interviews with locally-based partners who worked closely with the Ministry of Health and the NRL including WHO, CDC, Columbia University and the United States Agency for International Development (USAID).

To guide our review, we adapted an assessment protocol developed by WHO, CDC and USAID for use in IDSR programs in African countries [11]. Topics assessed included clarification of the disease surveillance goals and activities for the country, the role of the laboratory in surveillance activities, the coordination and structure of the laboratory network, and the collaboration of partners and resources for creating quality laboratory services for disease outbreak investigations and response.

## Results

### Surveillance goals and activities in the country

Rwanda's implementation of IDSR began in 2000 with a national assessment of the country's communicable disease surveillance program. The results of the assessment were used to develop a strategic plan for improving the country's surveillance, laboratory and response capacities. The WHO/AFRO IDSR technical guidelines were adapted to ensure that Rwandan disease priorities would be emphasized in the streamlined system. Training of health workers on IDSR approaches began in 2001. Additional training in laboratory and data management was provided for relevant staff through national and regional training workshops. The initial IDSR strategy in Rwanda targeted 19 priority diseases and syndromes (Table 1) recommended by WHO-AFRO because they are among the leading causes of illness, death and disability in African countries and are relevant to Rwanda based on epidemiologic criteria [3]. Implementation of the full IDSR program within the Ministry of Health is still under development; the focus of this review is on the laboratory component.

**Table 1:** Priority diseases and syndromes for Integrated Disease Surveillance and Response in Rwanda

Epidemic prone diseases and syndromes	Diseases targeted for eradication and elimination	Other diseases of public health importance
Cholera Bacillary dysentery Plague Yellow fever Meningococcal meningitis Vial hemorrhagic fever Severe acute respiratory syndrome (SARS) Avian influenza (AI)	Poliomyelitis Dracunculiasis Neonatal tetanus Leprosy	Diarrhea with dehydration in children less than 5 years of age Pneumonia in children less than 5 years of age HIV/AIDS Malaria Trypanosomiasis Tuberculosis Onchocerciasis

### The National Reference Laboratory

The Rwanda health service consists of a referral system encompassing three levels of health care: referral, district and health center levels. In the whole country, there are four referral hospitals, 34 district hospitals, and 385 health centers. At the central level, the NRL's mandate is to provide referral laboratory services to all health-care providers in the country, to prepare and distribute laboratory specimen transport media, to develop policies and to enforce standards for all laboratories in the country. The NRL also oversees the licensing, certification and accreditation of private and public health laboratories.

The NRL has autonomous status thus highlighting its role in promoting stronger laboratory capacities and leadership in disease control activities. Specific responsibilities of the NRL involve improved diagnostic capacities for HIV, tuberculosis, and malaria. The NRL also has capacity to identify and isolate bacterial causes of outbreaks. Additionally, the NRL's capacity to diagnose other diseases was expanded with support from USAID and CDC to diagnose influenza subtypes A and H5N1 as part of the country's epidemic preparedness and control activities.

Within the NRL, the laboratory personnel participate in investigations of suspected disease outbreaks in several ways. They ensure that specimen transport media is available. They also process and test specimens and then ship specimens to regional or international laboratories for further characterization as required by disease-specific guidelines. Other activities include training, supervision, and quality assessments for laboratories at all levels of the national health system.

At the time of this review, the Government of Rwanda was involved in decentralization of administrative functions that also impacted the health sector. Decentralization was seen as an opportunity for expanding laboratory capacity, improving data management and maximizing the use of surveillance information at all levels of the health care system in line with the IDSR strategy. In the Department of Epidemiology and Public Hygiene (DEHP), decentralization focused on improving coordination of disease surveillance with the alert and response system at the district level. Among the changes fostered by decentralization, a Geographic Information System (GIS) was set up, and bacteriology laboratories were established at five district hospital laboratories.

### Coordination and function of the laboratory network

The laboratory network in Rwanda is aligned with the overall three-tiered organization (central, district and peripheral levels) of the national health system. The network connects the central level's four reference hospital laboratories and the National Reference Laboratory with 34 district hospital laboratories at the intermediate level and 385 health center laboratories at the peripheral level.

Each level has a defined role supported by standard operating procedures (SOPs) that specify the safe collection, handling, storing, shipping and processing of laboratory specimens. Supervision and feedback are carried out using a level-specific checklist. The SOPs specify communication channels and procedures linking each level for the referral of laboratory specimens, reporting of data, supervision and quality assurance activities. For example, the district hospital laboratories submit weekly reports of selected priority diseases. The district level performs commonly requested laboratory tests such as hematology, clinical chemistry, urine analysis and parasitology. At the health center level, laboratories perform microscopy for detecting malaria, tuberculosis and other bacterial and parasitic agents. Almost all health center laboratories have the capacity to do human immunodeficiency virus (HIV) rapid testing. For some disease programs such as malaria, the network extends to the community level where volunteers are trained in detection and treatment of malaria including home-based management of malaria. The community volunteers contribute to surveillance activities by referring patients to health centers for laboratory services.

### Services at the National Reference Laboratory

The laboratory capacity for the isolation and identification of bacterial pathogens has been established at the NRL. Specimens for bacteria such as *Vibrio cholerae*, *Salmonella typhi*, *Shigella *species, *Neisseria meningitidis *and *Haemophilus influenzae *are collected from the peripheral levels and transported to the NRL where they are analyzed. The results are fed back to the districts and also shared with WHO/AFRO on a monthly basis.

Laboratory capacity for HIV is one of the most developed services in Rwanda, with major financial and technical support from the United States Government's President's Emergency Plan for AIDS Relief (PEPFAR) through CDC, other US Government organizations, the Mailman School of Public Health at Columbia University, the Global Fund, UNICEF and others. The NRL, located in Kigali, is equipped to perform the necessary biological tests required for the evaluation, treatment, and follow-up of HIV/AIDS patients including HIV rapid tests, viral load, and PCR. The NRL plays a leading role in organizing and setting up new HIV laboratory services (such as monitoring HIV treatment resistance), training, supervision, data management, and operational research.

The capacities for polymerase reaction (PCR), CD4 and viral load measurements have been available at the NRL since 2004. These capacities were expanded through collaboration with partners due to decentralization by providing services for fluorescence-activated cell sorting including FACS Count™ and FACS Calibur™ instruments. For example, two FACS Calibur™ instruments are at the NRL, 23 are in district hospitals and one is at a private research clinic in Kigali. At the end of 2005, the NRL performed nearly 70% of all CD4 counts conducted in the country. By 2007-2008, this figure from the NRL dropped to 36% due to increased capacity to conduct CD4 counts in 23 district hospitals. Plans are under way to upgrade the FACS™ Count instrument for assessing lymphocyte percentages for infants.

Decentralization of services was also implicated in expansion of access to antiretroviral treatment (ART) at the national level. Following the Rwanda National Paediatric Conference in 2004, there was recognition that early infant diagnosis of mother-to-child transmission (PMTCT) and ART sites should be expanded at all levels of the health system. In July 2005, with support from CDC, three paediatric diagnostic sites were established for early infant diagnosis using either dried blood spots (DBS) or whole blood methods. By April 2008, the number of rapid diagnostic sites had expanded to 126.

### Training of laboratory personnel

The strengthening of laboratory capacity including advocacy, provision of laboratory equipment, training and research was achieved through partner resources for specific tests and procedures. The training topics included advanced techniques for tuberculosis diagnosis (for example, testing for multi-drug resistance), HIV/AIDS testing (HIV serology, CD4 counts, and PCR for infant diagnosis of HIV), and techniques for hematology, biochemistry, and parasitology, including blood smears to detect malaria and intestinal helminths. Training topics also included good laboratory practices, bio-safety, biological sample collection, and the safe transport, handling and storage of specimens. In 2005, the NRL trained 467 biotechnologists on laboratory detection of malaria, TB, HIV rapid testing, standard laboratory practices and biosafety. In 2007, 969 laboratory personnel completed an integrated laboratory training in malaria, TB, and HIV (471 participants), biochemistry and haematology (61 participants) as well as training in how to perform CD4 counts (34 participants), conduct testing with dried blood spots (180 participants) and carry out HIV-specific testing at new VCT sites (223 participants).

### Supervision of laboratory activities within the national network

The national reference laboratory coordinates and carries out supervision for laboratory activities in referral hospitals, district hospitals, health centres and private clinics. The supervisory activities, which take place each trimester, are integrated across disease and subject areas and include: TB, malaria, HIV, biochemistry, and haematology. Supervision of bacteriology activities is carried out separately at referral and district hospitals only.

During the supervisory visits, a check list is used to assess performance in technical activities, the extent of compliance with SOPs, the quality and reporting of results, record keeping, internal quality controls, instrument performance (especially microscopes and spectrophotometers), availability and quality of reagents, and management of consumables. The extent of reliably available utilities (such as water and electricity), laboratory space and standard biosecurity capacities are also assessed. There are 420 facilities that receive at least one visit per year. Some may receive two or three visits during the year depending on the supervisory results. The number of actual supervisory visits to laboratory sites is maintained at a high level, e.g., 517 in 2005, 862 in 2006, and 689 in 2007. The supervisory visit data is analyzed and feedback is given to the respective health facilities. When results are not satisfactory, the NRL intervenes for corrective actions, in the form of retraining or amelioration of other causes of poor performance. Plans are underway to decentralize quality control and supervisory activities. In the new plan, the district hospital laboratories will take charge of the health centers while the NRL will supervise the referral hospitals, the district hospital and private laboratories.

### Collaboration with external laboratories

Disease specific programs in Rwanda are managed by the Government of Rwanda in collaboration with partners such as WHO, the Global Fund and PEPFAR. Mapping of health facilities ensures that each partner has specific facilities to support so that all facilities are aligned in support of national goals and objectives for control and prevention programs targeting HIV/AIDS, TB, malaria, epidemic disease surveillance and opportunistic infections. In this way, partner resources complement each other to fill the gaps in terms of sharing equipment, reagents, consumables, human resources and technical support. Merging of the HIV/AIDS, TB and malaria programs into one institution called the Treatment and Research AIDS Center (TRAC Plus) allows for clinical planning and laboratory activities in addition to collaboration at high levels. This coordination aims to avoid overlapping or duplication of activities in any area.

We observed examples of collaboration between national disease programs and the national and external reference laboratory systems such as those for polio, measles, and multi-disease resistant TB activities. For example, a key component in many WHO disease eradication or elimination programs (such as the polio eradication activity within the Expanded Programme on Immunization or EPI), is the collaboration between national disease programs and the national and external reference laboratory systems. In the case of polio eradication in Rwanda, the transport of stool specimens from patients with acute flaccid paralysis (AFP) originates at peripheral levels through district hospitals and then to the NRL or to the WHO-EPI program in Rwanda. The NRL or WHO-EPI program in turn ships these specimens to the Ugandan Virus Research Institute in Entebbe, Uganda (UVRI) for confirmation and characterization of the organism. The results are reported back to Rwanda's NRL and EPI offices and then shared with the WHO. The results are communicated to the district health team and health facilities that use the results to inform timely and relevant public health response actions.

For monitoring of the measles program, serum specimens from patients with suspected measles are collected and transported from the health facilities to the NRL. The samples are analyzed for viral antibodies by ELISA. The results are given to EPI for their use in determining a timely response. The results are shared with WHO-AFRO on a monthly basis. Serum specimens also are sent to UVRI on a quarterly basis for external quality control. In addition, the NRL receives quality control panels every quarter from UVRI.

Monitoring for multi-drug resistant TB is done by the NRL, and some specimens are subsequently referred to the external laboratory at the Institute of Tropical Medicine (IMTA) in Antwerp, Belgium for quality control (QC) testing. The QC panel samples for epidemic bacteria, malaria and TB microscopy, CD4 counts, HIV ELISA and Western Blot are received from the National Institute of Public Health (NIPH) in South Africa every quarter. The QC panels for assessing PCR capacity are received from CDC in Atlanta, USA.

### Laboratory results from the network

Table 2 illustrates examples of data produced through the laboratory network in support of the communicable disease surveillance system. Specimens reflected in the table are referred specimens or specimens collected by the national reference laboratory for confirmation of suspected cases during outbreak investigation or surveillance. Once specimens are confirmed according to criteria specified by WHO, there is no need for more specimens for laboratory investigations. Specimens continue to be processed to inform clinical management.

**Table 2:** Bacteriologic specimens and strains isolated by the National Reference Laboratory 2005 to 2007

		2005		2006		2007	
***Suspected outbreak***	***Specimen type***	***Number of specimens received***	***Number of confirmed isolates***	***Number of specimens received***	***Number of confirmed isolates***	***Number of specimens received***	***Number of confirmed isolates***

Cholera	Stool	46	3 *V. cholerae 01 *Ogawa 8 *V. cholerae 01 *Inaba	17	2 *V. Cholerae 01 *Ogawa	110	8 *V. cholerae 01 *Ogawa 1 *V. cholerae 01 *Hikojima

Dysentery	Stool	11	None	None	None	110 (same specimens as for cholera)	4 *E. coli *0157: H7 1 *Shigella flexneri *1 *Shigella sonnei*

Measles	Blood	188	0 measles 28 rubella	187	42 measles 23 rubella	132	12 measles 18 rubella

Typhoid fever	Blood	42	8 *Salmonella typhi* 1 *Salmonella. paratyphi B*	44	3 *Salmonella typhi* 1 *Staphylococcus aureus*	132	3 *Salmonella typhi* 1 *Staphylococcus aureus*

Meningitis	Cerebro-spinal fluid	20	2 *N. meningitidis* 5 *Streptococcus pneumoniae*	21	6 S*treptococcus pneumoniae*	22	6 *S. pneumoniae* 1 *S. agalactiae* 1 *C. neoformans* 1 *C. albudis*

The NRL conducts quality control (QC) and quality assurance (QA) activities for all levels of the network laboratories at regular intervals. The NRL also participates in the WHO-AFRO External Quality Assessment (EQA) scheme for enteric and meningitis pathogens, TB and malaria. External quality assurance for HIV, measles and selected bacterial diseases is carried out through designated international partner or disease-specific collaborating laboratories. As an example, in 2003, 100% concordance was reported for the unlinked, anonymous HIV testing in a total of 288 samples sent to CDC-Atlanta.

Health centres and district health facilities regularly send 10% of randomly selected HIV positive and 5% HIV negative specimens to the NRL for quality control assessment. The number of voluntary counselling and testing sites (VCT) participating in this QC program has increased yearly: there were 44 in 2003, 129 in 2004, 229 in 2005, 256 in 2006 and 313 in 2007. According to the 2007 annual report from the Rwanda Treatment and Research AIDS Center (TRAC), the number of sites for paediatric treatment (PMTCT) increased markedly between 2003 and 2007; there were 53 in 2003, 120 in 2004, 209 in 2005, 234 in 2006 and 285 in 2007. The QC results showed good performance with discordance rates decreasing over time: 3%, in 2003, 2.6% in 2004, 2% in 2005, 1% in 2007, and 0.8% in 2008. The NRL also collects 15 TB-slides per site per quarter for QC from health facilities (Center for Diagnosis and Treatment or CDT sites) in the country. The number of sites participating in QC and smear-positivity results from 2003 to 2007 is shown in Table 3.

**Table 3:** Quality control for TB slide examination from TB Diagnosis and Treatment Center (CDT) sites*

Year	Number of CDT sites participating in QC	Number of TB slides (specimens)	Percent (%) TB-smear positive	Percent (%) with false positive result	Percent (%) with false negative result	Overall performance
2003	60	No records available				

2004	134	No records available				

2005	147	3622	13.7%	6.4%	2.6%	Not rated

2006	173	7370	11.3%	4.1%	1%	Good

2007	183	9057	6.6%	2.7%	0.5%	Good

*The NRL was initiated in 2003 and organization of the network began in 2004. The lab network was able to begin reporting number of specimens by 2005. We attribute the gradual decrease from 13.7% to 6.5% to comprehensive training of laboratory technicians and to the national TB program in its goals for reducing spread of TB.

To ensure the quality of measles laboratory testing, each trimester the NRL sends 10% of the total measles and rubella samples received at the NRL to the Uganda Virus Research Institute (UVRI). The NRL also receives 20 panel specimens each year from the UVRI. The panels are tested and results are subsequently shared with UVRI. In 2004, the result showed good concordance and the NRL was subsequently accredited for measles laboratory confirmation by WHO/AFRO. In 2005, the Rwandan NRL obtained 95% concordance for measles and rubella testing.

### Challenges to the network

Documented reports and participant responses suggested that achieving a public health laboratory network is not without considerable challenges in major areas. For example, written reports and interviewee responses noted the following obstacles: a delay implementing an integrated strategy for improving an integrated public health surveillance and response strategy, insufficient number of skilled human resources, weaknesses in the general infrastructure, and shortages of vehicles, equipment, supplies, and reagents.

One of the major limitations cited by participants has been the delayed implementation of IDSR in Rwanda. Without a functional IDSR strategy in place, health staff reported that the role of laboratory is not well understood in its context of a broader public health surveillance network. While there is a strategic plan with defined goals for IDSR in place, implementation of the plan has yet to be fully implemented. At the time of this review, the surveillance and reporting system was using multiple forms from a variety of implementing partners resulting in a duplication of effort and resources.

The shortage of skilled human resources at all levels is due to high attrition and turnover of trained personnel. Decentralization has resulted in a gap in human resources for specialized testing at the national level and routine functions at the district and health facility levels. The long distances between some health centers and the national level - in addition to difficult driving conditions in some areas - contributes to a situation where specimens arrive at the NRL in inadequate condition (for example, hemolyzed samples for CD4 and measles IgM testing). Computer and internet access for transmitting data is affected by the erratic supply of electricity. Finally, participants described working within the context of ongoing shortages of vehicles, fuel, laboratory safety equipment, laboratory supplies and reagents.

## Discussion

The government of Rwanda, through collaboration with its partners, has begun to strengthen the organizational capacity of the public health laboratory network for identification and confirmation of priority diseases. The laboratory network has a hierarchal structure with the central referral laboratories as lead authorities and the health centre-based laboratories at the periphery. Other referral hospitals laboratories also link to the National Reference Laboratory. There is a defined role for each level with level-specific standard operating procedures. The NRL is the focal point for national public health laboratory leadership and coordination of partners to establish and expand the laboratory network. Reporting on specimens processed from the district and hospital laboratories is integrated and submitted along with the weekly disease reports for selected priority diseases. Supervision and feedback is done using a check list as applicable to the different levels.

The benefits of an organized laboratory network are evident in the increases in capacity and performance as illustrated in Tables 2 and 3. Table 2 illustrates an increase over time in the number of specimens received and isolates confirmed by the NRL between 2005 and 2007, and Table 3 highlights a 3-fold increase during the same time period in the number of health facility laboratories participating in quality control for TB microscopy.

The laboratory network in Rwanda is contributing to the successful strengthening of the disease surveillance and response system by ensuring involvement of the public health laboratory network in the planning for IDSR, laboratory participation in outbreak investigations, engagement in feedback communications, integration of laboratory indicators to monitor progress, and developing mechanisms for coordination of epidemic preparedness and response at all levels. The coordination and leadership role of the NRL and the commitment of multiple partners under its leadership provide unique opportunities for strengthening the laboratory networking in Rwanda.

Because a major focus in IDSR implementation is to strengthen the district level, the ongoing decentralization process provides a good opportunity for expanding IDSR implementation in all districts. Recently, a Field Epidemiology and Laboratory Training Program was established which will eventually result in considerable capacity building, especially if the applied training program is accompanied by a well- structured human resources plan for a career path through and retention of competent laboratory staff at all levels of the network.

The strengthening of the collaboration between the NRL and the Treatment and AIDS Research Center (TRAC Plus) through the Center for Communicable Disease Control in Rwanda will lead to improved coordination. As a first step, an integrated informatics system and a national integrated surveillance system are being developed, which will facilitate not only communication of data but also the sharing of results. A recommendation of the study team to the NRL was to consider implementation of international standards such as OECD's Global Laboratory Practices (GLP) [12] and to seek international accreditation.

## Conclusions

Recommendations for creating a functional public health laboratory network usually highlight the need for: (1) defined communication channels between the various levels of the health system, (2) explicit linkages with relevant Ministry of Health divisions, (3) a structure for coordination of laboratory activities, and (4) collaboration with funding and technical partners [5,7,8]. These elements of a laboratory network were evident in our review. For example, Rwanda's laboratory network benefits not only from government ownership and partner commitment but also from the defined structures and communication linkages between levels of the health system and with partners. Responsibility for coordination of laboratory activities through the NRL and TRAC Plus provides a structure for avoiding duplication of resources. The example of the collaboration between the government of Rwanda and its technical and funding partners in creating a functional laboratory network that provides results is one that can be emulated by other countries.

## Competing interests

The authors declare that they have no competing interests.

## Authors' contributions

HP, JN, JBG and SK conceived the study. PR, TM, JBG, SK and MHK organized and coordinated collection of field data. WA, JN, HP, OM and MHK guided the study design and coordinated the review. SK, JBG, MHK and OM analyzed data. HP and SK drafted the manuscript. HP finalized the manuscript. All authors read and approved the final manuscript.
